# A Prototype Sensor for In Situ Sensing of Fine Particulate Matter and Volatile Organic Compounds

**DOI:** 10.3390/s18010265

**Published:** 2018-01-18

**Authors:** Chee-Loon Ng, Fuu-Ming Kai, Ming-Hui Tee, Nicholas Tan, Harold F. Hemond

**Affiliations:** 1 Singapore-MIT Alliance for Research and Technology (SMART) Centre, 1 CREATE Way, CREATE Tower, #10-01, Singapore 138602, Singapore; minghui1991@hotmail.com (M.-H.T.); nicholastanyijian@hotmail.com (N.T.); 2 Agency for Science, Technology and Research (A*STAR), National Metrology Centre (NMC), 1 Science Park Drive, Singapore 118221, Singapore; kai_fuu_ming@nmc.a-star.edu.sg; 3 Parsons Laboratory, Massachusetts Institute of Technology, Room 48-425, 15 Vassar Street, Cambridge, MA 02139, USA; hfhemond@exchange.mit.edu

**Keywords:** volatile organic compounds sensor, fine particulate matter sensor, in situ real-time air quality sensor, optical sensor, multi-pass absorbance, scattering

## Abstract

Air pollution exposure causes seven million deaths per year, according to the World Health Organization. Possessing knowledge of air quality and sources of air pollution is crucial for managing air pollution and providing early warning so that a swift counteractive response can be carried out. An optical prototype sensor (AtmOptic) capable of scattering and absorbance measurements has been developed to target in situ sensing of fine particulate matter (PM2.5) and volatile organic compounds (VOCs). For particulate matter testing, a test chamber was constructed and the emission of PM2.5 from incense burning inside the chamber was measured using the AtmOptic. The weight of PM2.5 particles was collected and measured with a filter to determine their concentration and the sensor signal-to-concentration correlation. The results of the AtmOptic were also compared and found to trend well with the Dylos DC 1100 Pro air quality monitor. The absorbance spectrum of VOCs emitted from various laboratory chemicals and household products as well as a two chemical mixtures were recorded. The quantification was demonstrated, using toluene as an example, by calibrating the AtmOptic with compressed gas standards containing VOCs at different concentrations. The results demonstrated the sensor capabilities in measuring PM2.5 and volatile organic compounds.

## 1. Introduction

Air pollution is a major environmental problem causing over seven million premature deaths every year [1]. The main air pollutants include criteria pollutants (i.e., particulate matter, carbon monoxide, ozone, nitrogen dioxide, sulfur dioxide, and lead) listed in the National Ambient Air Quality Standards [2] and other hazardous air pollutants (e.g., benzene, toluene, ethyl benzene, formaldehyde, naphthalene, acetaldehyde, trichloroethylene, and tetrachloroethylene) emitted from outdoor sources (e.g., industrial facilities, refineries, gas stations, vehicles, and wildfires) and indoor sources (e.g., building, construction materials, upholstered furniture and wood products, and fuel combustion for cooking or heating) [2,3,4]. To protect public health and to control emission sources, criteria pollutants and some of the hazardous air pollutants are usually monitored by conventional networks of static and sparse air quality monitoring stations [2]. Advancement towards smaller and portable sensing technologies at lower cost with capabilities to detect critical pollutants could greatly improve both temporal and spatial air pollution monitoring networks [5,6,7,8,9].

Common gas sensing technologies include optical methods, gas chromatography, and electrochemical approaches (e.g., metal-oxide semiconductors, polymers, carbon nanotubes) [5,10]. The optical method is one of the promising approaches because of its high accuracy, strong sensitivity, low environmental interference (e.g., temperature, humidity), and longer lifetime [6,7,10]. In this work, we developed a novel optical sensing system (AtmOptic) to detect fine particulate matter and some common hazardous gases (e.g., toluene) utilizing optical scattering and the multipass absorbance principle of sensing, respectively. Scattering is a physical process whereby some forms of radiation (e.g., light) are forced to deviate from a straight trajectory by one or more paths due to localized non-uniformities (e.g., particulate matter) in the medium through which they pass. The multipass absorbance method is used to measure weak spectra in gases or liquids by multiplying the effective path length through a medium and thereby increase absorbance.

## 2. Materials and Methods

### 2.1. Instrument

**Figure 1:**
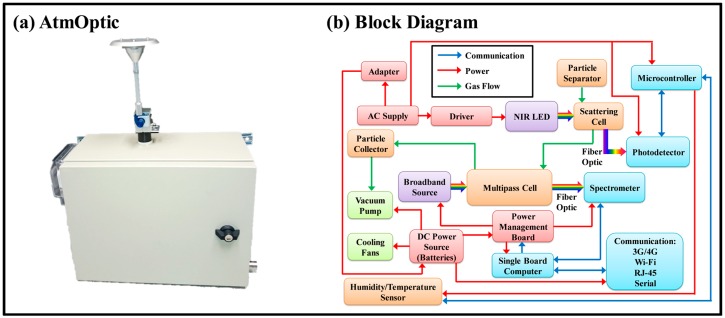
(**a**) Front views of the AtmOptic packaged inside a 40 × 15 × 30 cm enclosure for fixed location sensing; (**b**) Block diagram of the AtmOptic.

The layout of the AtmOptic is shown in Figure 1. The optical functions of the AtmOptic rely on the combination of two custom-designed flow cells, a scattering cell fitted with a light-emitting diode (LED) of 780 nm wavelength for particulate matter (PM) measurements using nephelometry and a broadband (185 to 1100 nm) light source that illuminates a multipass cell constructed with two concave mirrors for volatile organic compound (VOC) measurements. Flow into the cells is via an inlet fine particle-sorting cyclone and a pathway that contains a rectangular bend to minimize the entrance of stray light. The flow passed through the scattering cell, multipass cell, and a particle collector before being released back to the atmosphere. Light from the scattering cell is observed with an amplified photodetector (Thorlabs, Newton, NJ, USA, Model PDF10A/M) and the data are recorded with an in-house microcontroller made with an Arduino UNO running custom software (pAtmOptic). Light from the multipass cell is observed with a spectrometer (Ocean Optics, Dunedin, FL, USA, USB4000) and the data are recorded with a single-board computer manufactured by Technologic systems (Fountain Hills, AZ, USA, Model TS-7260-64-128F) running custom software (iLEDLIF) developed by [11,12]. For continuous monitoring, a dual AIRPO (sgbotics, Singapore, Model S2028B) vacuum pumps draws air samples into the cells manifold. Particles are collected with Millipore^®^ glass-fiber filters (Merck Millipore, Sigma-Aldrich, Singapore, Type 5, Lot 3110) and the humidity is measured with a temperature-corrected humidity sensor (ANSAC, Singapore, Model 104100-00). 

### 2.2. Fine Particulate Matter

**Figure 2:**
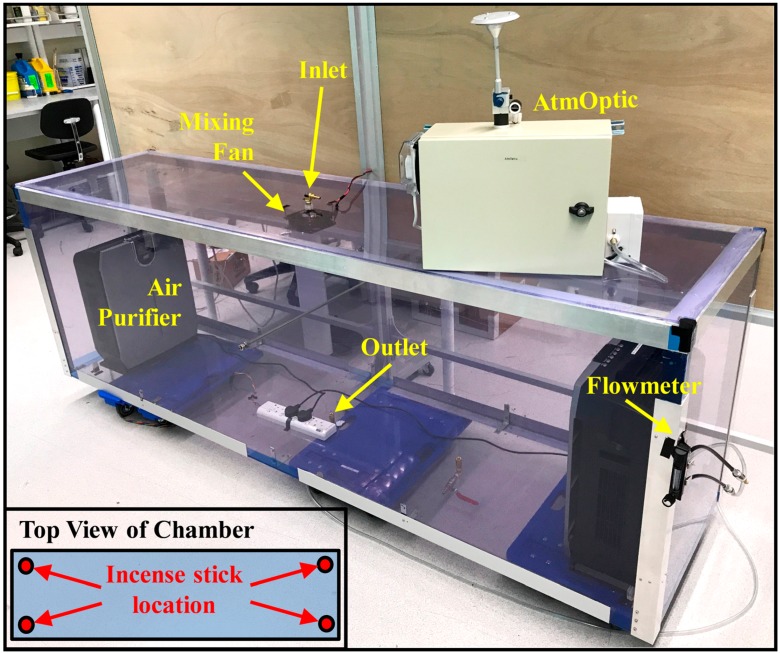
Experimental setup to study AtmOptic scattering response to PM2.5 concentration produced by burning incense sticks. Insert schematic shows the PM sources (incense sticks) locations.

The experimental setup of the AtmOptic scattering response to fine particulate matter (PM2.5) concentration is shown in Figure 2. PM2.5 refers to particulate matter of 2.5 µm or less in diameter. A 2.35 × 0.63 × 0.82 m in-house test chamber constructed with plexiglas reinforced with an aluminum frame was used to calibrate the AtmOptic for PM2.5 sensing. The AtmOptic continuously drew an air sample from an outlet centered at the base of the chamber. The flow rate is measured with a Key Instruments Polycarbonate flowmeter (RS, Singapore, Model MR3A14BVBN). Ambient air entered the chamber through an inlet centered at the lid of the chamber. An incense stick (旺里香) purchased from a local store in Singapore was burned at the four corners (insert graph in Figure 2), and the emissions produced were mixed by an Ebmpapst fan (element 14, Singapore, Model 4650X) instrumented below the inlet of the chamber to promote the mixing of smoke particles to achieve homogeneity. Each sampling cycle consists of a 30 min background air monitoring period (*t* < 0), the combustion period (0 ≤ *t* ≤ *T*), and a 60 min post-burning period (*t* ≥ *T*); following the experimental procedure of [13]. The averaged concentration (i.e., mass per volumetric of air) of PM2.5 covering the combustion and post-burning period of each run was computed by measuring the mass of the Millipore*^®^* glass-fiber filter with a Mettler Toledo XP6 (Cole-Parmer, Vernon Hills, IL, USA) microbalance before and after the mass collection for each run and the volumetric flow rate through the AtmOptic.

The chamber was thoroughly cleaned with damp disposable paper towel after each run. Two American Air Filter (AAF) air purifiers (AAF Singapore, Singapore, Model PurAir 400A) equipped with six layers of High Efficiency Particulate Air (HEPA) filtration technology capable of covering a total area of 80 to 120 m^3^ were used to remove air particles inside the chamber for 25 min after each run. The background air in the chamber was checked with the AtmOptic after the cleaning and the percent difference in the baseline signals were found to never exceed 1.7%. For all experiments, the background air signals in the chamber were monitored for 30 min and used for baseline correction and the flow rate was maintained at 1.5 L/min throughout the experiment with a regulated control.

For instrument comparison, the PM2.5 air sample measured by the AtmOptic was passed into the Dylos air quality monitor (Qtech Integrated, Singapore, Model DC1100 Pro) for particle counting before it was collected with the glass-fiber filter for mass measurement. It is not feasible to quantify the exact properties of each microscopic particle being counted by the Dylos. Therefore, it was assumed that (1) all particles are spherical with a density of 1.65 × 10^12^ µg/m^3^ [14]; (2) the radius of the particle in the PM2.5 channel is 0.44 µm [15]; and (3) 0.01 ft^3^ is converted to m^3^ by multiplying the factor 3531.5. The mass of a PM2.5 particle can then be computed as 5.89 × 10^−7^ µg and the PM2.5 concentration ($C_{PM2.5}$) can be obtained using:

(1) $$C_{PM2.5}=3531.5\left(No.\;of\;Particles\right)\left(5.89\times 10^{-7}\right)$$

Dylos was chosen for comparison because it was found to compare reasonably well with the air management system (AMS) in Philadelphia [16]. 

### 2.3. Volatile Organic Compounds

All experiments were performed using ultra high purity air (Leeden National Oxygen Ltd., Singapore) as the reference gas. The VOC vapor produced by the aqueous solution or solid state source was continuously drawn into the AtmOptic by the vacuum pump of the sensor when it was placed near the sensor inlet. For cargenogenic VOC (e.g., Benzene), the experiment was performed inside a fumehood (VEC Environment Pte Ltd., Singapore, Model Dynaflow GRP). For VOC (i.e., toluene in synthetic air) and ultra-high-purity air in compressed gas tanks, the gas was transferred from the tank to the inlet of the AtmOptic via tubing. After testing, the gas from the AtmOptic was exhausted to the fumehood through tubing. The AtmOptic was purged with ultra-high-purity air after each run to ensure the signal returned to the baseline before the next experiment.

#### 2.3.1. Laboratory Chemicals

Eight VOC spectra (vapor phase) were recorded using the AtmOptic: (1) Acetone; (2) Benzene; (3) Methanol; (4) Naphthalene; (5) *O*-xylene; (6) Phenol; (7) Styrene; and (8) Toluene. Acetone (CAS 67-64-1, Item No. A1084-1-4000) was obtained from Orëc (Duga Products and Services Co., Ban Suan, Chongburi, Thailand), benzene (CAS 71-43-2, Lot l219765525, Prod. 100515F) was from VWR International (Singapore), methanol (CAS 67-56-1, Product No. 179337, Lot SHBH4599V) was from Sigma-Aldrich (Singapore), naphthalene (CAS 91-20-3, Lot A0266833) was from Acros Organics (Fisher Scientific, Singapore), *O*-xylene (CAS 95-47-6, Lot 1413783) was from Sigma-Aldrich (Singapore), phenol (CAS 108-95-2, Lot l14Y009) was from Alfa-Aesar (Fisher Scientific, Singapore), styrene (CAS 100-42-5, Lot STBF-4819V) was from Sigma-Aldrich (Singapore), and toluene (CAS 108-88-3, Lot SHBF-4825V) from Sigma-Aldrich (Singapore). Compressed gas tanks containing different concentrations of toluene in synthetic air (Leeden National Oxygen Ltd., Singapore) from the BTEX (benzene, toluene, ethylbenzene, and *O*-xylene) group were used to demonstrate the quantification of VOC with the AtmOptic and the same calibration procedure can be repeated for other chemicals of interest.

#### 2.3.2. Household Products

The VOC spectra of various household products were recorded using the AtmOptic: (1) Elmer’s extra strong spray adhesive (Product No. E455); (2) 7CF interior/exterior spray paint (Color 39 black, Product No. R-8088); (3) Innisfree eco nail remover; (4) Araldite epoxy (5 min rapid cure); (5) Brasso metal polish; and (6) Gas lighter fluid. The test was performed by pouring an arbitrary amount of the products into a separate container and placing it near to the inlet of the AtmOptic for measurement.

## 3. Results and Discussion

### 3.1. Fine Particulate Matter

**Figure 3:**
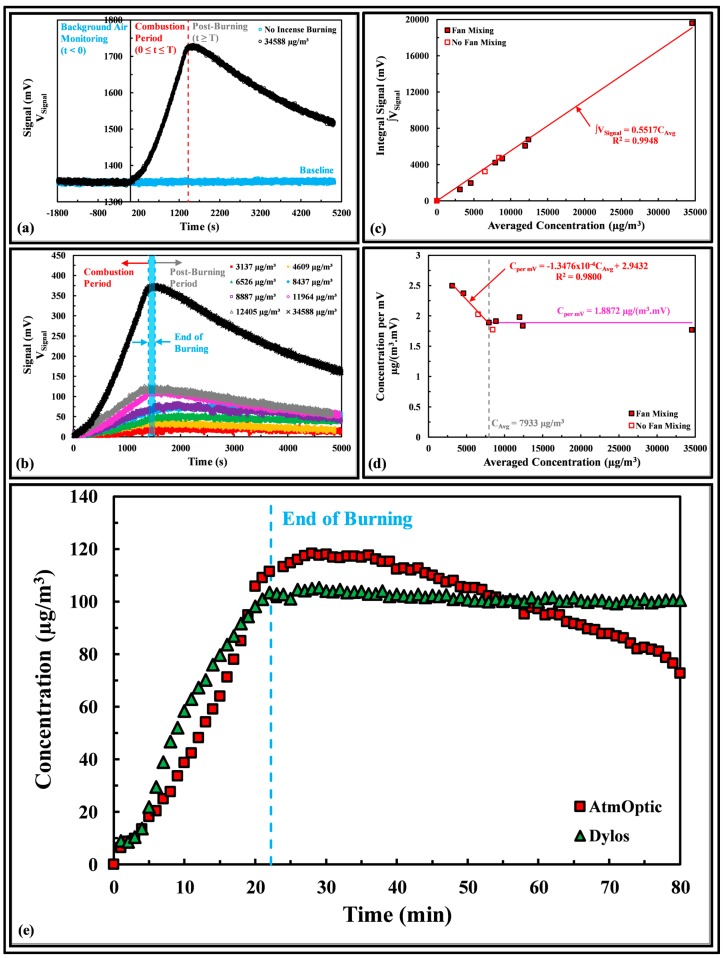
(**a**) Background air qualification and measured signal of seven incense sticks burning as a function of time; (**b**) Measured signal of the averaged concentration of PM2.5 produced by burning various numbers of incense sticks as a function of time. Legend: averaged concentration of PM2.5; (**c**) Integral signal as a function of averaged concentration of PM2.5, with and without fan mixing; (**d**) Concentration per millivolt as a function of the averaged concentration of PM2.5, with and without fan mixing; (**e**) Instruments comparison for PM2.5 measurements at an averaged concentration of 8511 µg/m^3^.

Figure 3a shows that the background signal at the baseline was stable when there is no incense burning and the PM2.5 (if any) in the ambient was constant in the absence of the incense smoke. During the combustion period (0 ≤ *t* ≤ *T*), the PM2.5 in the tank started to increase and the scattering signal rose to the maximum at the end of the combustion period. Post-burning, the signal began to drop over time as particle decay was observed. Figure 3b shows that the peak maximum of the PM2.5 signal increases with the amount of burned incense. The end of the burning time varies within 126 s (blue shaded region) for all sampling cases. Once the incense sticks were lit in the chamber, the PM2.5 concentration increased rapidly until the last incense stick burned out at *t* = *T*. The averaged concentration of PM2.5 corresponded to the time covering both the combustion and post-burning periods. Figure 3c shows that the integral signal is a linear function of the averaged concentration. There was no apparent adverse effect on the two measurements obtained without fan mixing. Figure 3d shows that the concentration per millivolt [$C_{per\;mV}$, in µg/(m^3^·mV)] of the AtmOptic decreases linearly with the increase in averaged concentration ($C_{Total}$) when it is less than 7933 µg/m^3^:

Uncorrected:

(2) $$C_{per\;mV}=-1.3476\times 10^{-4}C_{Avg}+2.9432$$

Thereafter, the concentration per millivolt remained constant at *C_per mV_* = 1.8872 µg/(m^3^·mV). 

Figure 3e compares a PM2.5 measurement of the AtmOptic with the Dylos. The total mass of PM2.5 collected by the filter was 1.05 mg over a volume of 0.1234 m^3^ computed based on a constant flow rate of 1.5 L/min, resulting in an averaged concentration of 8511 µg/m^3^. Both instruments appeared to capture a similar emission profile with a small variation in concentration at a given time. Unlike AtmOptic, the Dylos observed a constant concentration closed to 100 µg/m^3^ post-burning and particle decay was not observed by the instrument. By applying the trapezoidal rule [$\left(t_{2}-t_{1})(\frac{C_{2}-C_{1}}{2}\right)$; where $t_{1}$ is previous time step, $t_{2}$ is current time step, $C_{1}$ is previous concentration value, and $C_{2}$ is current concentration value] to approximate the area under the curve and using the observed constant flow rate of 1.5 L/min, the total mass of PM2.5 computed with the data of both instruments were 0.88 mg, a 16.2% lower than the that (1.05 mg) collected by the filter. Relative humidity (RH) could be a factor causing the discrepancy because the experiments were conducted around a RH between 50% and 70%. Day et al. found that aerosol light scattering measurements were a function of RH, particularly for RH values above 60% [17]. 

### 3.2. Volatile Organic Compounds

**Figure 4:**
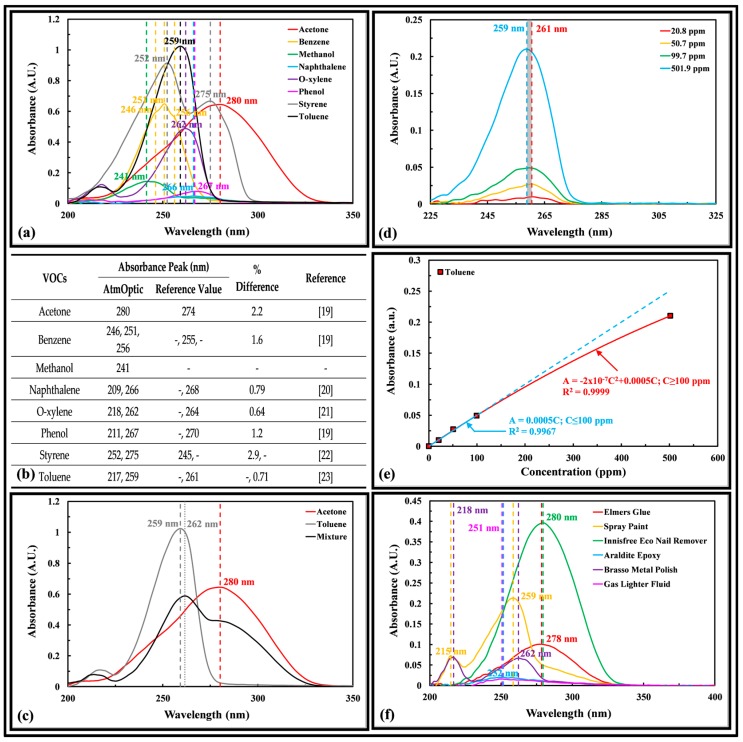
(**a**) Absorbance spectra of various VOCs measured with the AtmOptic; (**b**) Comparison of the AtmOptic absorbance peak with various references [19,20,21,22,23]; (**c**) Absorbance spectra of an acetone and toluene mixture; (**d**) Absorbance spectra of toluene of different concentrations; (**e**) Toluene absorbance peaks as a function of concentration; (**f**) Absorbance spectra of various household products.

Figure 4a shows the absorbance of different VOC peaks at different wavelengths, demonstrating that it is possible to detect and classify different VOCs using the multipass absorbance method in the AtmOptic. Figure 4b compares the absorbance peak of various VOCs with published values and found them to be in very good agreement. Figure 4c shows that the absorbance of toluene in a toluene and acetone gas mixture peaks at 262 nm, a red shift of 3 nm relative to a pure toluene gas, which may be associated with the function of admixture concentrations. The acetone absorbance in the mixture peaks at 280 nm and remained unchanged relative to a pure acetone gas. Exposure to this gas mixture may be encountered in a chemical manufacturer facility that produces a ready-to-use acetone–toluene solvent mixture. Figure 4d shows the absorbance spectra of toluene in a synthetic air peak between 259 and 261 nm. Figure 4e shows that the absorbance peak obeys the Beer–Lambert [18] equation [A=εlC, where A is absorbance (absorbance unit), ε represents absorptiviity, l denoted optical path length, and C is concentration] up to 100 ppm. Each data point in the graph is an average of five measurements. Figure 4f shows that the absorbance spectra of different VOCs’ presence in various household products peaks at different wavelengths, demonstrating the potential specificity of the AtmOptic in detecting these products. Both the Elmer’s glue and Innisfree eco nail remover contain acetone showing an absorbance peak at 278 and 280 nm, respectively. The 7CF interior/exterior spray paint contain toluene that peaks at 219 and 259 nm. The Araldite epoxy peaks at 252 nm. The Brasso metal polish peaks at 218 and 262 nm. The gas lighter fluid contains butane and peaks at 251 nm. 

## 4. Conclusions

The results described illustrate the AtmOptic’s capabilities in measuring both the PM2.5 and VOCs present in the air, and suggest that it is possible to identify and quantify fine particulate matter and various VOCs using the optical method. When fully characterized, the AtmOptic can be used for sensing transboundary haze, VOC discharge from industry, and VOCs in consumer products and gas stations, among others. Matrix effects and changes in environmental conditions in these measurements were not investigated in this paper, which could be important and subsequently studied. For larger particles (e.g., PM10), scattering increases in the forward direction and decreases in the backward (light-facing) direction due to constructive and destructive interference. The exact relationship can be established through calibration using similar procedure as for PM2.5 and the replacement of a PM10 cyclone in the AtmOptic. Finally, a spectrum separation algorithm can be applied to improve the quantification of VOCs.
